# The prognostic significance of TSPO-PET imaging in IDH-mutant glioma: a single-center, retrospective study

**DOI:** 10.1007/s00259-026-07926-y

**Published:** 2026-05-30

**Authors:** Katharina J. Müller, Roman Stürzl, Sabrina Kirchleitner, Isabelle von Polenz, Viktoria Ruf, Veit M. Stoecklein, Jonas Reis, Stefanie Quach, Lena Kaiser, Julia Lorenz, Adrian Zounek, Patrick N. Harter, Rainer Rupprecht, Markus J. Riemenschneider, Matthias Brendel, Niklas Thon, Joerg-Christian Tonn, Louisa von Baumgarten, Nathalie L. Albert

**Affiliations:** 1 Department of Neurology, LMU University Hospital, LMU Munich, Munich, Germany; 2 Department of Nuclear Medicine, LMU University Hospital, LMU Munich, Munich, Germany; 3 Department of Neurosurgery, LMU University Hospital, LMU Munich, Munich, Germany; 4 Center for Neuropathology and Prion Research, Faculty of Medicine, LMU Munich, Munich, Germany; 5 German Cancer Consortium (DKTK), Partner Site Munich, a Partnership Between DKFZ and LMU University Hospital, Munich, Germany; 6 Institute of Neuroradiology, LMU University Hospital, LMU Munich, Munich, Germany; 7 Department of Neurosurgery (Evangelisches Klinikum Bethel), Medical School, Bielefeld University, Bielefeld, Germany; 8 Department of Neuropathology, Regensburg University Hospital, Regensburg, Germany; 9 Bavarian Cancer Research Center (BZKF), Munich, Germany; 10 Department of Psychiatry and Psychotherapy, Molecular Neurosciences, University of Regensburg, Regensburg, Germany; 11 German Center for Neurodegenerative Diseases (DZNE) Munich, Munich, Germany; 12 Munich Cluster for Systems Neurology (SyNergy), Munich, Germany

**Keywords:** IDH-mutant glioma, TSPO-PET, Imaging, Neuroinflammation, Prognostication

## Abstract

**Purpose:**

Clinical prognostication and decision-making in IDH-mutant glioma is increasingly complex, especially with new targeted treatment options like IDH-inhibitors. Individual patient risk stratification for better treatment planning is needed; however, standard prognostic models rely on clinical and histologic parameters as well as MRI, which may not fully reflect the tumor’s biological behavior. Positron emission tomography (PET) imaging of the 18 kDa translocator protein (TSPO) is known as surrogate marker of activated microglia and macrophages and enables non-invasive assessment of the tumor microenvironment and peri-/intratumoral inflammation as well as TSPO-positive tumor cells. The aim of this study was to investigate TSPO-PET imaging in IDH-mutant glioma and its association with outcome.

**Methods:**

In this monocentric, retrospective study, 46 patients with newly diagnosed IDH-mutant glioma who had undergone TSPO-PET imaging with [¹⁸F]GE180 prior to any therapeutic intervention were included. Quantitative PET parameters including mean and maximum standardized uptake values (SUV_max_, SUV_mean_) and the respective PET-positive tumor volumes were evaluated for their association with clinical data and time to next intervention (TTNI), and overall survival (OS).

**Results:**

The cohort consisted of 27 patients (58.7%) with astrocytoma, IDH-mutant (median age 36 years (30–51)) and 19 patients (41.3%) with oligodendroglioma, IDH-mutant and 1p/19q-codeleted (median age 41 years (36–48)). High SUV_max_ on TSPO-PET imaging was associated with shorter TTNI, and OS (*p* = 0.0118 and *p* = 0.0459, respectively). In multivariate analyses adjusting for age, KPS, WHO grade, FET-PET-positive volume, and tumor volume on contrast-enhanced MRI, the TSPO-PET-positive volume was associated with TTNI (hazard ratio (HR) = 1.037, 95% CI: 1.009–1.064, *p* = 0.0106).

**Conclusions:**

This study highlights the potential prognostic utility of TSPO-PET imaging in newly diagnosed IDH-mutant glioma. Our findings support the inclusion of PET imaging in future clinical trials to develop imaging-based risk models for better prognostication and individualized treatment guidance.

**Graphical abstract:**

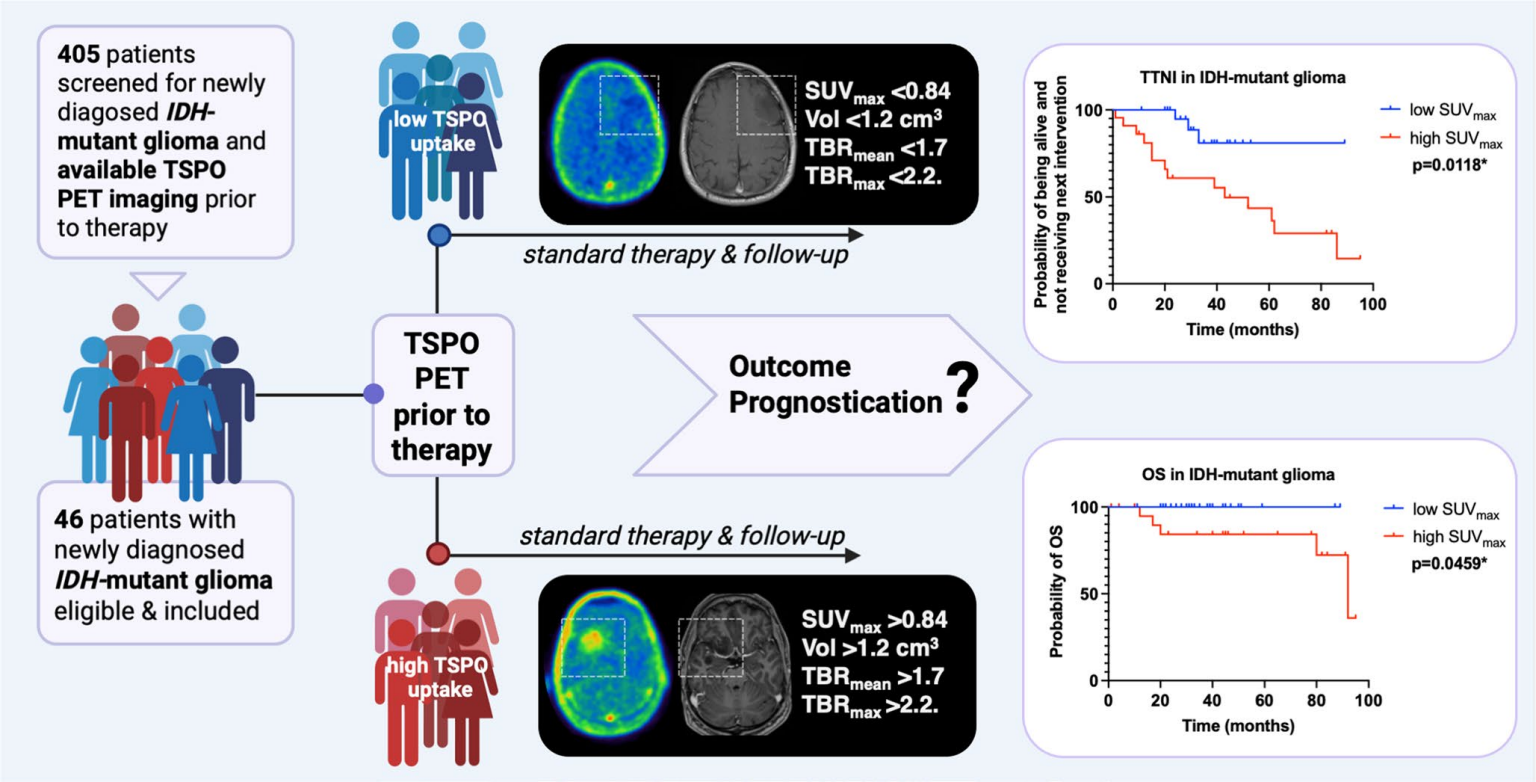

**Supplementary Information:**

The online version contains supplementary material available at 10.1007/s00259-026-07926-y.

## Introduction

Diffuse gliomas with isocitrate dehydrogenase (IDH) mutations represent a biologically distinct subgroup of adult brain tumors, encompassing astrocytoma, IDH-mutant and oligodendroglioma, IDH-mutant and 1p/19q-codeleted [1, 2]. IDH-mutant gliomas, typically affecting younger adults, progress more slowly and have better outcomes than IDH-wildtype glioblastomas, but remain incurable [3, 4]. Postoperative management must be carefully tailored, weighing options such as radiochemotherapy, systemic therapy, or active surveillance based on clinical risk factors for recurrence. A watch-and-wait strategy may be appropriate in low-risk patients, while those with advanced age, subtotal resection, neurologic deficits or higher-grade histology often receive radiochemotherapy [3]. The wide range of potential treatment options and their side effects make treatment decisions challenging, and the aim is to balance oncologic control with long-term neurotoxicity (particularly in this younger patient population). Therefore, more accurate and validated clinical biomarkers to guide risk-adapted treatment in this molecularly defined subgroup are needed [4]. The recent advent of targeted therapies such as vorasidenib, along with a growing interest in immunomodulatory strategies in clinical trials, underscore the demand for personalized risk stratification models [5, 6].

In this context, positron emission tomography (PET) has emerged as a valuable tool for assessing the metabolic tumor burden and changes as a complement to magnetic resonance imaging (MRI) [7]. Amino acid PET using tracers like [¹⁸F]FET or [¹¹C]Methionine can identify metabolically active tumor regions independently of the integrity of the blood-brain barrier leading to more accurate tumor delineation [8, 9]. However, amino acid PET predominantly reflects tumor metabolism and does not capture the full complexity of the tumor microenvironment. Growing evidence suggests that tumor-associated macrophages and microglia (TAMs) significantly influence glioma progression and therapy resistance [10, 11]. The 18 kDa translocator protein (TSPO), expressed in the outer mitochondrial membrane of activated microglia and infiltrating immune cells, serves as a promising target for imaging neuroinflammation [12, 13]. TSPO-PET imaging, particularly with high-affinity ligands like [^18^F]GE180, enables visualization of inflammatory responses and tumor-host interactions beyond the structural and metabolic changes captured by MRI and amino acid PET [14]. However, TSPO is expressed not only by TAMs but also by the tumor cells themselves, especially in glioblastoma [11, 14]. While the exact cellular sources and their relative contributions to the TSPO signal are not yet fully understood, elevated TSPO expression and tracer uptake has been linked to increased tumor aggressiveness and resistance in IDH-wildtype glioblastoma [15–18]. Previous studies have indicated that TSPO tracer uptake correlates with tumor progression and recurrence in IDH-wildtype glioblastomas, yet, its prognostic value in newly diagnosed IDH-mutant glioma remains unclear [17]. This study aimed to investigate the prognostic value of TSPO-PET imaging in newly diagnosed IDH-mutant glioma, focusing on and discussing its utility for clinical risk stratification and prognostication. By integrating TSPO-PET data with other established imaging and molecular markers, we seek to advance the personalized management of this biologically distinct glioma subtype in an era of expanding therapeutic options.

## Methods

### Study design and patient selection

**Fig. 1:**
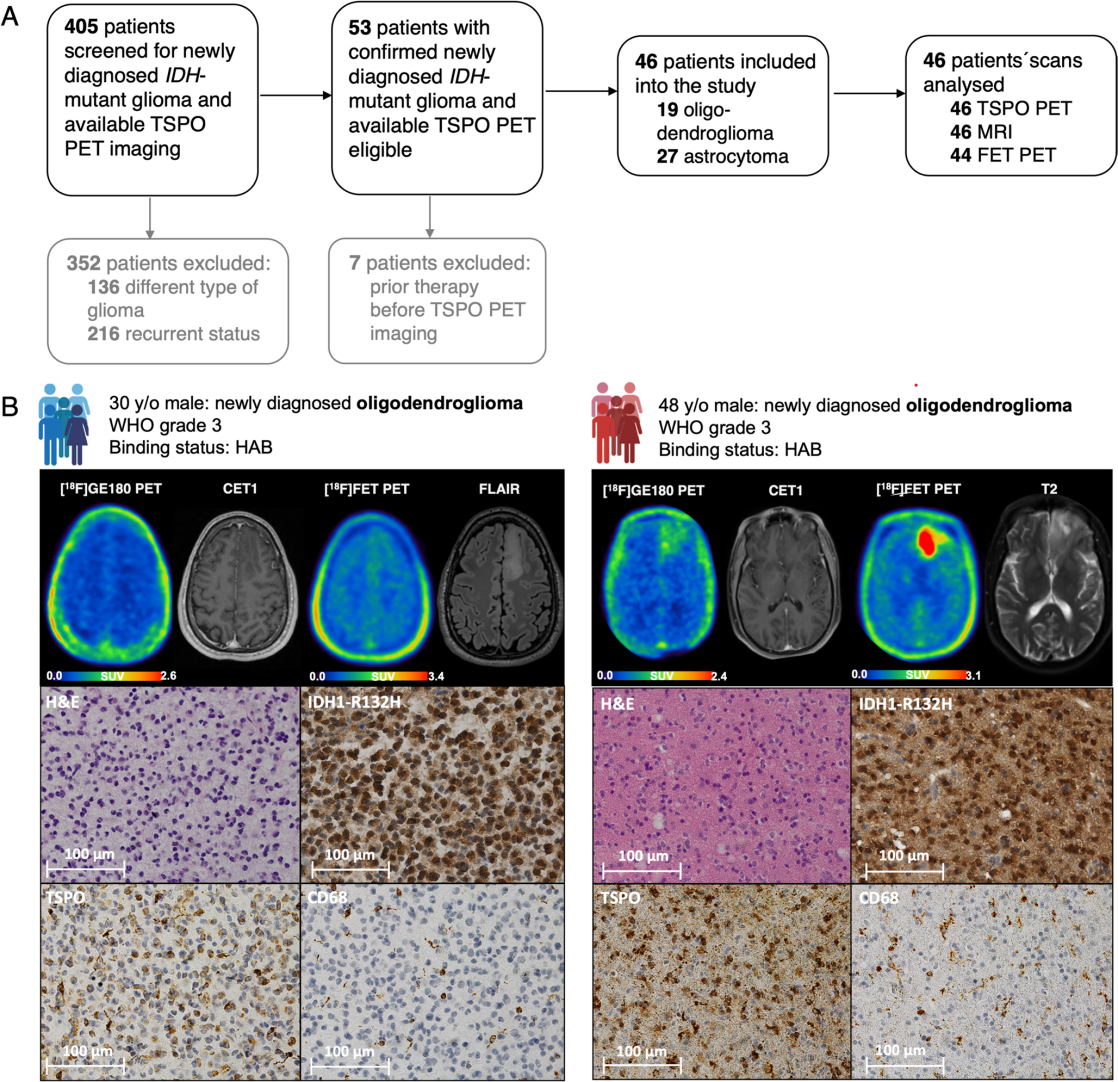
(**A**) Patient selection flow chart from our PET imaging database (**B**) Representative images of an IDH-mutant glioma case with low and high TSPO tracer uptake on [¹⁸F]GE-180-PET, shown with corresponding histological and immunohistochemical analyses. Hematoxylin and eosin (H&E) staining revealed comparable overall cell density, with slightly lower values in the high-uptake case. IDH1-R132H immunohistochemistry demonstrated a comparable tumor cell density in both cases. TSPO and CD68 immunohistochemistry showed weak and scattered TSPO expression and few CD68-positive cells in the low-uptake case. In contrast, the high-uptake tumor exhibited numerous strongly TSPO-positive cells and slightly higher number of CD68-positive tumor-associated macrophages. Scale bars are included in the images (magnification×400)

In this monocentric, retrospective study, a total of 46 patients with histologically confirmed newly diagnosed IDH-mutant glioma with available [^18^F]GE-180-PET and standard MRI prior to therapeutic interventions were identified and included (Fig. 1A). In addition, a concomitant [^18^F]FET-PET was conducted in 44 patients prior to radiochemotherapy. Patients had undergone either stereotactic biopsy or microsurgical tumor resection according to standard clinical procedures. Histological and molecular genetic assessments followed established protocols, with all cases classified as IDH-mutant glioma according to the 2021 World Health Organization’s Classification of Tumors of the Central Nervous System [1]. All histological diagnoses were made by board-certified neuropathologists. Imaging analysis was conducted by a set of independent investigators (KJM, NLA, IvP, JR) according to the conventional MRI-based RANO 2.0 and to the PET-based PET RANO 1.0 criteria (for amino acid PET) [19, 20].

### Immunohistochemistry and hematoxylin and eosin staining

Immunohistochemistry (IHC) and Hematoxylin and Eosin (H&E) staining were performed on formalin-fixed, paraffin-embedded (FFPE) tissue sections as previously described [21]. IHC was conducted manually using the EnVision™ Detection Kit (K4065, Dako). Heat-induced epitope retrieval (HIER) was performed for 30 min in 10 mM citrate buffer (pH 6.0) for CD68 staining, and in 1mM Tris/EDTA buffer (pH 9.0) for TSPO and IDH1-R132H staining. The following primary antibodies were used: anti-TSPO (1:5000, clone: EPR5384, ab109497, Abcam), anti-CD68 (1:200, clone: PGM1, M0876, Dako), and anti-IDH1-R132H (1:20, clone: H09, DIA-H09, Dianova). Detection was carried out using DAB as chromogen, followed by counterstaining with hematoxylin.

### TSPO and FET-PET imaging analysis

All PET scans were acquired using a Biograph 64 PET/CT scanner (Siemens, Erlangen, Germany). Tracer synthesis and image acquisition were performed as previously described. For [¹⁸F]GE-180 PET, approximately 185 MBq of [¹⁸F]GE-180 were administered as a single intravenous bolus. Summation images from 60 to 80 min post-injection were used for analysis. For [¹⁸F]fluoroethyl-L-tyrosine (FET)PET, a dose of approximately 185 MBq [¹⁸F]FET was administered intravenously. Summation images from 20 to 40 min post-injection were analyzed. As no histologically validated threshold for [¹⁸F]GE180-PET imaging has yet been established, we applied a background-based segmentation approach analogous to the standard method used for glioma delineation in [¹⁸F]FET-PET [22]. Background activity was defined as the mean signal intensity of at least six crescent-shaped cortical regions in the contralateral healthy hemisphere. To minimize potential confounding effects from treatment or background inflammatory activity, the primary imaging measure in [¹⁸F]GE180-PET imaging was maximal tumor uptake, quantified as the maximum standardized uptake value (SUV_max_), as reported earlier [17]. We also calculated the tumor-to-background ratios (TBR_max_) by dividing the SUV_max_ by the background mean, following EANM/EANO/RANO guideline recommendations for amino acid PET imaging and as also reported previously for [¹⁸F]GE180-PET imaging [22, 23]. The PET-positive volume was semi-automatically delineated using a standardized threshold of 1.6 times background activity, as previously validated (institutional program: Hermes Medical Solutions, Inc., Stockholm, Sweden).

### MRI analysis

Routine MRI included contrast-enhanced T1-weighted (CE-T1w), and non-enhanced T1- and T2-weighted (T2w) sequences. According to RANO 2.0 criteria, tumor extension was quantified as the sum of the products of the largest axial diameter and its perpendicular diameter (SPD) for each selected target lesion, measured separately for contrast-enhancing and, when appropriate, non-enhancing T2/FLAIR components (institutional program: Visage Imaging, Inc., San Diego, CA) [20].

### Statistical analysis

Results are presented as median values with interquartile ranges (IQR). The association between categorical variables was assessed using the chi-square (χ²) test, with results presented as absolute frequencies and percentages. Continuous variables were tested for normality and homogeneity of variances using the D’Agostino–Pearson test. Nonparametric group comparisons were performed using the Mann-Whitney U test. Spearman’s correlation coefficient was used to test for correlation between two continuous variables. Time to next intervention (TTNI), as introduced as a secondary endpoint in the INDIGO trial, was defined as the primary outcome measure and the interval from first surgery (or biopsy) to either the first treatment for recurrence/progression or last follow-up. For patients without a documented intervention, the imaging date prompting a treatment decision was used. Overall survival (OS) was defined as the interval from first surgery (or biopsy) until death or last follow-up. Survival analyses were conducted using the Kaplan-Meier method, with group differences assessed via the log-rank test. Thresholding was performed using median splits for all variables. Multivariate survival analysis was performed via Cox proportional hazard models. The Cox model included possible influencing variables known from the literature, such as age, WHO grade (2 vs. 3–4), KPS and the competing tumor volumes on TSPO-PET, FET-PET, and MRI. A p-value ≤ 0.05 was considered statistically significant. All statistical analyses were carried out using Prism (version 10.5.0, GraphPad Software Inc., San Diego, CA). The graphical abstract was created using biorender.

### Ethics approval

All patients provided written informed consent prior to [^18^F]GE180 or [^18^F]FET-PET/CT scans which were performed as part of the clinical routine. The study was conducted in accordance with the Declaration of Helsinki and followed Good Clinical Practice guidelines. Approval was granted by the local Ethics Committee (Institutional Review Board Nos.: 18–783 and 17–656).

## Results

The study involved a cohort of 46 patients with newly diagnosed IDH-mutant glioma, which included 19/46 (41.3%) patients with oligodendroglioma, IDH-mutant and 1p/19q-codeleted (median age 41 years (36–48), 12/19 patients, 63.2% male and 7/19 patients, 36.8% female) and 27/46 (58.7%) patients with astrocytoma, IDH-mutant (median age 36 years (30–51), 16/27 patients, 59.3% male, 11/27 patients, 40.7% female) (Table 1). Median Karnofsky performance status (KPS) was 100% (90–100) in oligodendroglioma and 90% (90–100) in astrocytoma (Table 1). A total of 12/19 of oligodendroglioma patients (63.2%) and 18/27 of astrocytoma patients (66.6%) received initial microsurgical resection. First-line treatment after surgical intervention (resection or biopsy) was heterogenous with 2/19 (10.5%) oligodendroglioma patients and 5/27 (14.8%) astrocytoma patients underwent a watch-and-wait strategy, whereas 7/19 (36.8%) oligodendroglioma patients and 8/27 (29.6%) astrocytoma patients received combined radiochemotherapy postoperatively (Table 1).

**Table 1:** Patient characteristics of the study cohort

Patient characteristics *n* = 46			Oligodendroglioma *n* = 19		Astrocytoma *n* = 27			*p*-value
Age (median, IQR)			41 (36–48)		36 (30–51)			0.134
Sex (number, %)								0.961
Male			12 (63.2)		16 (59.3)			
Female			7 (36.8)		11 (40.7)			
KPS (median, IQR)			100 (90–100)		90 (90–100)			0.429
WHO grade (number, %)								
2			8 (42.1)		14 (51.9)			
3			11 (57.9)		10 (37.0)			
4			-		3 (11.1)			
Mode of initial surgical intervention (number, %)								
Biopsy			7 (36.8)		9 (33.3)			
Resection			12 (63.2)		18 (66.6)			
First-line postoperative treatment (number, %)								0.551
Observation			2 (10.5)		5 (14.8)			
Radiochemotherapy (PC)			7 (36.8)		8 (29.6)			
Radiotherapy only			6 (31.6)		12 (44.4)			
Other (Experimental, [^125^I] seeds)			4 (21.1)		2 (7.4)			
*MGMT* promoter methylation status (number, %)								0.632
Methylated			18 (94.7)		21 (77.7)			
Non-methylated			1 (5.3)		4 (14.8)			
Partially methylated			0		2 (7.4)			
*TERT* promoter mutation status (number, %)								
Mutant								*0.001
C250T mutation			3 (15.8)		0			
C228T mutation			15 (78.9)		1 (3,7)			
Wild-type			0		26 (96.3)			
Not available			1 (5.3)		0			
TSPO Binding Status (number, %)						0.781		
HAB			9 (47.4)		11 (40.7)			
MAB			5 (26.3)		9 (33.3)			
LAB			2 (10.5)		2 (7.4)			
Not available			3 (15.8)		5 (18.5)			

PC *=* procarbazine; *MGMT* = *O*^6^-alkylguanine DNA methyltransferase gene; *TERT* = telomerase reverse transcriptase gene; KPS = Karnofsky performance status; HAB = High-affinity binding status. MAB = Medium-affinity binding status; LAB = Low-affinity binding status. Qualitative data are number and percentage; continuous data are median and IQR. Percentages may not total 100 due to rounding. Categorical variables were analyzed using the chi-square (χ²) test. Group comparisons of continuous variables were assessed with the Mann–Whitney U test

Molecular subtypes are given in Table 1: The *MGMT* promotor was methylated in 18/19 patients (94.7%) in the oligodendroglioma and in 21/27 patients (77.7%) in the astrocytoma group (Table 1). *TERT* promoter mutations were significantly more frequent in oligodendroglioma (18/19 patients, 94.7%) with C228T being the most common mutation (78.9%), while 26/27 patients (96.3%) were wildtype in astrocytoma. TSPO binding affinity status was determined for 38/46 patients (82.6%): 4 (10.5%) patients were low-affinity binders, 14 (36.8%) medium-affinity binders, and 20 (52.6%) high-affinity binders (Table 1). To illustrate the relationship between TSPO-PET signal intensity and underlying histopathological features, we analyzed representative cases of IDH-mutant gliomas with low and high TSPO tracer uptake on [¹⁸F]GE-180-PET using H&E staining as well as IHC for IDH1-R132H, TSPO and CD68 (Fig. 1B). H&E staining revealed comparable overall cell density between both cases, with slightly reduced values in the high-uptake tumor. IDH1-R132H IHC confirmed a comparable tumor cell density (> 80%) in both cases. TSPO IHC showed that the low-uptake tumor exhibited weak and scattered expression, whereas the high-uptake tumor displayed numerous strongly TSPO-positive cells. CD68 IHC further demonstrated a slightly increased number of CD68-positive tumor-associated macrophages in the high-uptake case, suggesting that both tumor and immune cells contribute to the elevated TSPO-PET signal (Fig. 1B).

### TSPO-PET metrics and imaging characteristics

Tumor volume delineation on CE-T1w MR images showed a median volume of 0.27 cm^3^ (0-0.7 cm^3^) in oligodendroglioma and 0 cm^3^ (0-1.5 cm^3^) in astrocytoma, whereas tumor volume delineation on T2w images revealed a median volume of 16 cm^3^ (11–29 cm^3^) in oligodendroglioma and 20 cm^3^ (9.7–37 cm^3^) in astrocytoma, which was not significantly different (Table 2).

**Table 2:** Multimodal imaging characteristics

Imaging characteristics	Oligodendroglioma (WHO grade 2/3)	Astrocytoma (WHO grade 2/3/4)	*p*-value
MRI	*n* = 19	*n* = 27	
CE-T1w volume [cm^3^]	0.27 (0-0.7)	0 (0-1.5)0.903	
T2w volume [cm^3^]	16 (11–29)	20 (9.7–37) 0.311	
Presence of CE (number, %)	10 (52.6)	10 (37.0) 0.941	
[^18^F]GE-180-PET	*n* = 19	*n* = 27	
Background activity	0.39 (0.36–0.45)	0.38 (0.35–0.41)0.402	
SUV_mean_	0.65 (0.6–0.9)	0.65 (0.51–0.80) 0.584	
SUV_max_	0.69 (0.65–1.6)	0.84 (0.56–1.1) 0.285	
TBR_mean_	1.71 (1.62–2.12)	1.71 (1.42–2.01) 0.679	
TBR_max_	2.11 (1.71–3.11)	2.2 (1.5–2.82) 0.385	
PET positive volume [cm^3^]	0.42 (0.14-11.0)	2.0 (0.21-8.0) 0.671	
[^18^F]FET-PET	*n* = 18	*n* = 26	
Background activity	0.91 (0.82–1.02)	1.1 (0.88–1.1) 0.056	
SUV_mean_	1.9 (1.43–2.13)	1.6 (1.21–2.03) 0.162	
SUV_max_	2.8 (1.7–4.22)	2.01 (1.5–2.61) *0.036	
TBR_mean_	2.0 (1.75–2.12)	1.71 (1.03–1.9) *0.012	
TBR_max_	2.9 (1.81-4.0)	1.92 (1.41–2.8) *0.005	
PET positive volume [cm^3^]	12.01 (0.52–25.2)	1.41 (0-7.3) *0.005	

CE-T1w = contrast-enhanced T1-weighted; T2w = T2-weighted; [^18^F]GE-180 = Flutriciclamide; [^18^F]FET = ^18^F-fluoroethyl-L-thyrosine. PET = positron emission tomography; SUV_mean_ = mean standardized uptake values; SUV_max_ = maximal standardized uptake values; TBR_mean_ = mean target-to-background ratio; TBR_max_ = maximal target-to-background ratio. Continuous data are median and IQR. Group comparisons of continuous variables were assessed with the Mann–Whitney U test

Quantification of [^18^F]GE-180-PET metrics revealed no significant differences between oligodendroglioma and astrocytoma (Table 2). [^18^F]GE-180-PET metrics did also not differ significantly between different affinity binding status (MAB/LAB vs. HAB) (all metrics not significant; *n* = 18 and *n* = 20, respectively) (Supplemental Table 2).

Quantification of standard [^18^F]FET-PET imaging parameters, including background activity, SUV_mean_, SUV_max_, TBR_mean_, TBR_max_, and PET-positive volume, showed significant differences between the two groups (Table 2): there were significant differences with higher SUV_max_ (2.8; 1.7–4.22; *p* = 0.036), TBR_mean_ values (2.0; 1.75–2.12; *p* = 0.012), and TBR_max_ values (2.9; 1.81-4.0; *p* = 0.005) in oligodendroglioma compared to astrocytoma (median SUV_max_ 2.01 (1.5–2.61), median TBR_mean_ 1.71 (1.03–1.9), and median TBR_max_ 1.92 (1.41–2.8)). Moreover, the FET-PET-positive volume differed significantly between the two groups with higher tumor volumes in oligodendroglioma (*p* = 0.005, Table 2).

**Fig. 2:**
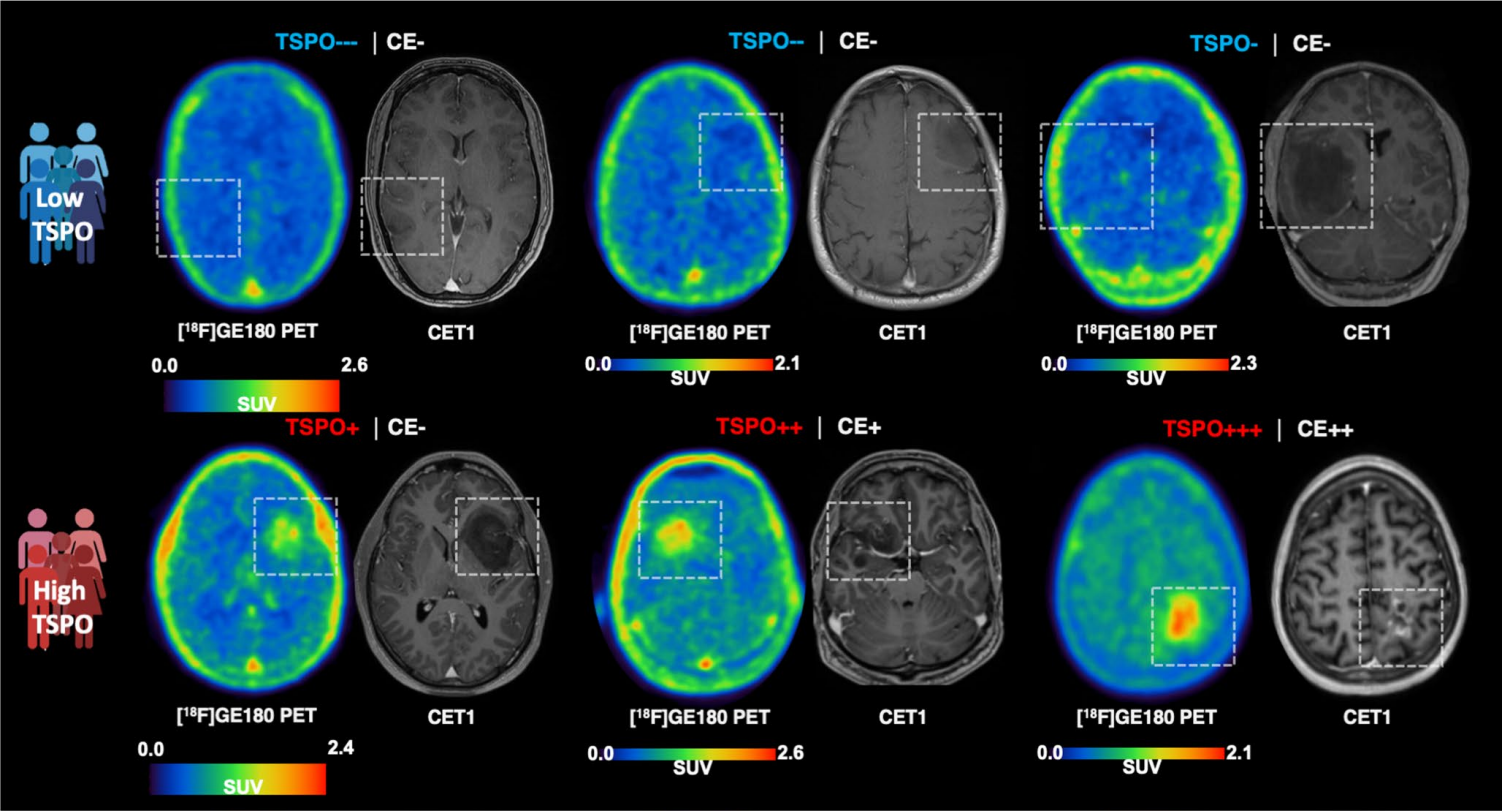
Representative TSPO-PET images alongside CE-T1w MRI. Two groups are depicted, one with low TSPO tracer uptake and absent CE (indicated in blue in the first row) and the other with high TSPO tracer uptake with either absent or present CE on MRI (indicated in red in the second row)

**Fig. 3:**
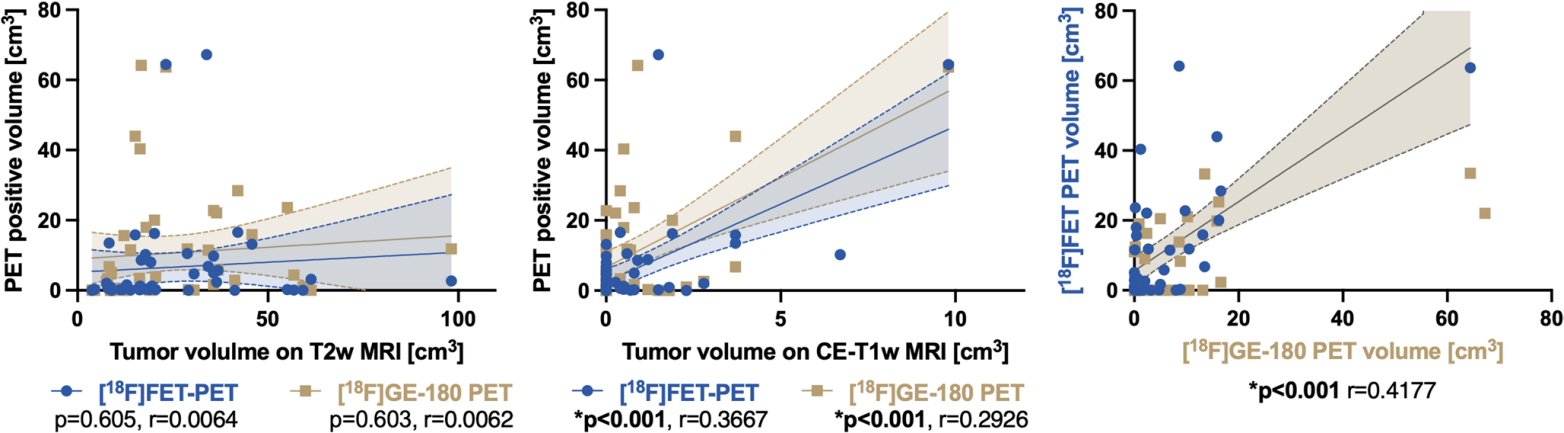
Tumor volume on T2w MRI showed no significant correlations with either [¹⁸F]FET-PET (*p* = 0.605) or [¹⁸F]GE-180 PET (*p* = 0.603). By contrast, volumes derived from CE-T1w MRI were significantly correlated with both [¹⁸F]FET-PET (*p* < 0.001) and [¹⁸F]GE-180 PET (*p* < 0.001). Additionally, the [¹⁸F]FET-PET–positive volume was associated with the volume on [¹⁸F]GE-180-PET (*p* < 0.001)

Correlational analysis between multimodal imaging techniques revealed significant associations (Figs. 2 and 3). The tumor volume measured on T2w MRI showed no significant correlation with either [^18^F]FET-PET (*p* = 0.605) or [^18^F]GE-180-PET (*p* = 0.603). In contrast, the tumor volume on CE-T1w MRI demonstrated a significant correlation with both [^18^F]FET-PET (*p* < 0.001) and [^18^F]GE-180-PET (*p* < 0.001). Moreover, [^18^F]FET-PET-positive volume was strongly correlated with [^18^F]GE-180-PET-positive volume (*p* < 0.001, Fig. 3).

### Association with clinical data and outcome

**Fig. 4:**
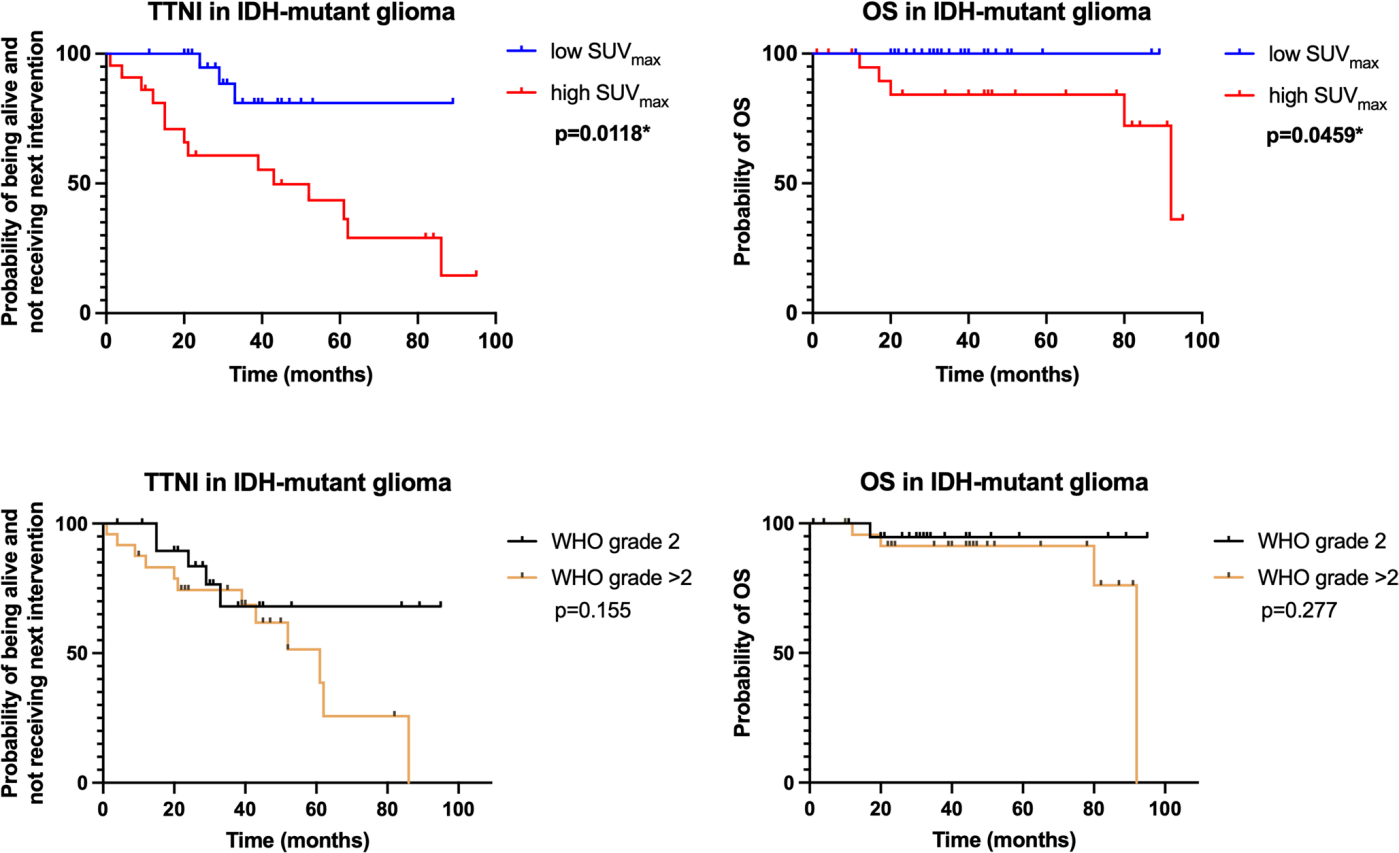
Kaplan–Meier analysis of TTNI and OS stratified by TSPO-PET SUV_max_ and WHO grade (*n* = 46). Higher SUV_max_ was significantly associated with shorter TTNI and OS (*p* = 0.0118 and *p* = 0.0459), whereas WHO grade showed no significant association (*p* = 0.155 and *p* = 0.277)

Median TTNI was 61 months (95% CI: 43 to 91), and median OS was 91 months (95% CI: 22 to not estimable) in the total cohort. Kaplan-Meier estimates for TTNI and OS were not significantly different based on WHO grade (*p* = 01550, *p* = 0.2769 Fig. 4). However, TTNI and OS showed significant differences, when comparing high vs. low tracer uptake based on TSPO-PET metrics (Fig. 4 and Supplemental Fig. 1). With TSPO-PET imaging, we identified significant associations between low vs. high SUV_max_ and both TTNI and OS (*p* = 0.0118 and *p* = 0.0459). When comparing secondary parameters, such as low vs. high TBR_mean_, we also observed associations with TTNI (*p* = 0.0216), and OS (*p* = 0.0377), as well as for TBR_max_ and TTNI (*p* = 0.0118). Based on the TSPO-PET-positive volume, TTNI and OS were significantly different (*p* = 0.0012, and *p* = 0.0309, Fig. 4). For survival analysis based on FET-PET, we also found a significant association between high FET-PET-positive tumor volume and TTNI (*p* = 0.0378, Supplemental Fig. 2). However, there was no significant association with any outcome metrics based on other FET-PET-parameters, such as TBR_mean_ or TBR_max_ (Supplemental Fig. 2). Further, no significant outcome differences were observed, when stratified for the presence of CE on MRI in our univariate analysis (Supplemental Fig. 2). When analyzing the association between [¹⁸F]GE180-PET parameters and TTNI separately for WHO grade 2 (*n* = 22) versus 3–4 (*n* = 24), we observed that in WHO grade 2 tumors a larger [¹⁸F]GE180-PET-positive volumes was significantly associated with shorter TTNI (*p* = 0.0038, Supplemental Fig. 3). In contrast, in WHO grade 3–4 tumors, increased TBR_mean_ was significantly associated with reduced TTNI (*p* = 0.0455). Compared with WHO grade 2 tumors, WHO grade 3–4 gliomas showed significantly higher CE-T1 volumes (*p* = 0.001), increased TBR_max_ and volume on [¹⁸F]FET-PET (*p* = 0.006, *p* = 0.004 respectively), and higher TBR_max_ and volume on [¹⁸F]GE-180 (all *p* = 0.008, *p* = 0.003 respectively), while T2 volume did not differ between groups (*p* = 0.938, Supplemental Table 1).

**Fig. 5:**
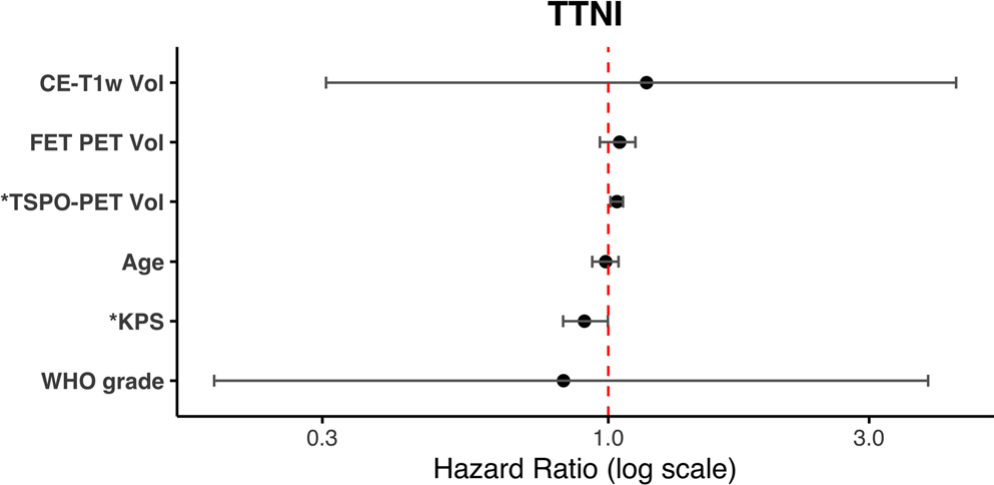
Multivariate cox proportional hazard ratio analysis of potential influencing variables for TTNI. Patients with higher TSPO-PET-positive volume had an increased risk of earlier intervention (HR = 1.037, 95% CI: 1.009–1.064, *p* = 0.0106). We also found a significant association between higher KPS and prolonged TTNI (HR = 0.9045, 95% CI: 0.8268–0.9982, *p* = 0.0462). In contrast, FET-PET–positive volume, age, WHO grade, and tumor volume on CE-T1w MRI showed no significant association with TTNI in this cohort. Multivariate OS analysis was not feasible due to a limited number of events

Multivariate Cox proportional hazards model revealed a significant association between TSPO-PET-positive volume and shorter TTNI (Fig. 5). Patients with greater TSPO-PET-positive volume showed an increased hazard of requiring earlier intervention (hazard ratio (HR) = 1.037, 95% CI: 1.009–1.064, *p* = 0.0106) (Fig. 5). However, FET-PET-positive volume and tumor volume on CE-T1w MRI were not associated with TTNI in this cohort (HR = 1.049, 95% CI: 0.9653–1.121, *p* = 0.2242; HR = 1.175, 95% CI: 0.3047–4.327, *p* = 0.8090 respectively), nor was age, or higher WHO grade. Further, a significant relationship was observed between higher KPS and prolonged TTNI (HR = 0.9045, 95% CI: 0.8268–0.9982, *p* = 0.0462). Multivariate outcome analysis of OS was not feasible in our model due to the relatively short surveillance time and only 4 events registered so far.

## Discussion

Our study investigated the prognostic value of [^18^F]GE-180-PET imaging in a well-defined cohort of newly diagnosed IDH-mutant glioma and suggests its potential utility as a non-invasive imaging biomarker for clinical risk stratification. Our key findings demonstrate that the TSPO-PET-positive volume is significantly associated with shorter TTNI, independent of conventional clinical factors such as age, WHO grade, and aggressive tumor characteristics on MRI, such as CE-volume. These findings suggest that [^18^F]GE-180-PET imaging may reflect tumor activity beyond metabolic tumor delineation on FET-PET or tumor dimension on CE-T1w MRI. TSPO-PET imaging therefore could serve as an early prognosticator and indicator for risk stratification and treatment planning.

Our findings align with and extend prior evidence indicating that TSPO-PET imaging was associated with glioma progression and therapeutic resistance and previous studies have also demonstrated that TSPO tracer uptake (SUV_max_) is indicative of tumor progression in recurrent IDH-mutant gliomas [24]. Although the cellular sources of TSPO expression need to be disentangled further, published data suggests that TSPO expression is heterogeneous in glioblastoma and only partly driven by tumor-associated macrophages and activated microglia, while tumor cells themselves had a greater impact on the TSPO signal [14, 24, 25]. The observed prognostic correlation between large tumor volume on TSPO-PET and poor outcomes supports the concept that baseline tumor-associated TSPO signal and peri- and intratumoral neuroinflammatory activity may indicate a more aggressive disease course even in molecularly favorable IDH-mutant glioma subtypes [24, 26, 27].

Our data suggest that different imaging modalities depict complementary tumor components: although tumor delineation via CE-T1w MRI, [^18^F]GE-180-PET volume and [^18^F]FET-PET volume are correlating, they seem to capture different aspects of glioma biology [26, 28]. TSPO-PET-positive signal was associated with worse outcomes in our study cohort, independent of CE on MRI or FET-PET-positive volume. This discrepancy raises questions regarding the distinct biological processes underlying metabolic tumor activity versus tumor-associated TSPO-signals and/or intra-/peri-tumoral inflammation and supports a multimodal imaging approach for comprehensive tumor assessment [29]. Notably, the contrast-enhancing tumor volume on MRI did not significantly correlate with outcomes in our cohort, highlighting the limitations of conventional imaging in low-proliferative IDH-mutant gliomas [30]. Nevertheless, MRI remains the standard imaging modality for clinical assessment according to the RANO 2.0 criteria [20]. Further, FET-PET metrics did not correlate with survival in our cohort, which may be due to the small sample size. Additionally, the known higher FET uptake typically seen in oligodendrogliomas has likely contributed to the lack of observable survival differences as these tumors generally are associated with better prognosis [31].

In low-proliferative tumors, subtle yet clinically meaningful changes may occur independently of blood–brain barrier disruption and can be missed on conventional MRI, as for example only a fraction of patients had morphologic response on MRI during the INDIGO trial [29, 31]. Therefore, metabolic imaging with amino acid PET is increasingly being used and discussed as potentially valuable prognostic biomarker and imaging biomarker for treatment monitoring in patients receiving IDH-inhibitor therapy [31, 32]. Also, histopathologic WHO grading in IDH-mutant gliomas can be heterogeneous with great inter-observer variability of ill-defining boundaries between tumor grades, which may limit the predictive value of histopathological grading alone [33]. In contrast, PET uptake intensities provide standardized quantitative measurements that capture the entire tumor volume rather than relying on a selected tissue samples from biopsy material, which may partly explain why imaging biomarkers can demonstrate prognostic associations independent of WHO grade. A recent study by *Mair et al.* supports the prognostic value of FET-PET and showed that high FET-PET signal in newly diagnosed IDH-mutant gliomas has shown an association with worse outcome, also independently from WHO grade [34]. The same association was not found in our study but may be related to the circumstance that FET-PET signal associations with outcome need to be analyzed separately for astrocytomas and oligodendrogliomas due to the well-known significantly differing uptake characteristics of both tumor entities which results in a very limited sample size in our study [31]. In TSPO-PET we could not find significant differences regarding uptake intensity between the two subgroups. Therefore, TSPO-PET imaging could be of complementary use by reflecting different aspects of tumor biology, particularly interesting and valuable in clinical trials involving immunotherapeutic agents [35]. Stratifying patients based on TSPO tracer uptake into low-risk (low uptake) and high-risk (high uptake) IDH-mutant glioma or capturing changes of the tumor microenvironment under therapy could help identify biological responses during immunotherapy that precede radiographic progression [36]. Hypothetically, ‘high-risk’ patients with high TSPO-PET-positive volume may warrant more intensive monitoring or earlier intervention. This approach may be especially relevant with the introduction of targeted therapies like IDH-inhibitors, where early identification of biologically active disease could optimize treatment timing and patient selection [6].

Despite the insights gained from our study several limitations need to be acknowledged. First, the small sample size and limited number of outcome events, particularly for OS, constrain the generalizability of our findings. Second, TSPO binding affinity genotyping was incomplete in a subset of patients. TSPO binding affinity status reflects a genotype polymorphism (rs6971) that could be a possible confounder influencing PET signal intensity. However, the impact of the polymporphism is still not clear for TSPO-PET imaging in neuro-oncological patients: in a previous study of mixed cohorts (neuro-oncological and neurodegenerative diseases) a potential impact of the rs6971 polymorphism could be shown with slightly lower TSPO signals in regions of healthy background in LAB patients, however in more recent studies no significant differences in tumoral tracer uptake could be detected in glioblastoma patients based on binding status [36, 37]. In line with these findings, we could not detect any significant differences of tumoral tracer uptake when comparing genotype variants our study. TSPO binding status did not differ significantly between tumor types, nor did it significantly influence TSPO-PET metrics. These findings support the feasibility of [^18^F]GE-180 PET quantification across binding affinity groups in clinical practice. Thirdly, the cellular sources contributing to the TSPO-PET signal in IDH-mutant glioma remain insufficiently defined due to the lack of histological correlation, limiting precise clinicopathological interpretation. The signal measured with [¹⁸F]GE-180-PET likely reflects a composite of several factors, including TSPO expression in tumor cells and microglia. However, tracer uptake may also be partly influenced by blood–brain barrier disruption and tumor-associated vascular changes, which can contribute to the measured signal independently of cellular TSPO expression [38]. Consequently, [¹⁸F]GE-180 may not represent the optimal tracer for isolating TSPO-specific effects in glioma tissue. Newer-generation TSPO tracers with improved brain penetration may help overcome these limitations and warrant further investigation in future studies [39]. Finally, the observational nature of the study limits causal inference, and prospective validation in larger, multi-center cohorts is warranted. Further studies and longitudinal imaging data are needed to evaluate how TSPO and FET-PET metrics evolve over time or if TSPO-PET can predict progression earlier than FET-PET or MRI.

## Conclusion

This study shows the potential of [^18^F]GE-180-PET imaging as a prognostic biomarker in newly diagnosed IDH-mutant glioma. Although it remains unclear to which extent [^18^F]GE-180-PET-positivity reflects tumor-associated macrophages and/or TSPO-PET-positive tumor cells, the negative association of TSPO-PET-signal with patient’s outcome suggests that this imaging approach may help identify ‘high-risk’ IDH-mutant glioma cases and provide valuable prognostic insights to support more accurate clinical decision-making. Our findings warrant further investigation to evaluate the role of TSPO-PET in imaging-based risk stratification.

## Supplementary Information

Below is the link to the electronic supplementary material.

Supplementary Material 1

Supplementary Material 2

Supplementary Material 3

Supplementary Material 4

## Data Availability

Data are available on qualified request from the corresponding author.

## References

[1] Louis DN, Perry A, Wesseling P, Brat DJ, Cree IA, Figarella-Branger D, et al. The 2021 WHO Classification of Tumors of the Central Nervous System: a summary. Neuro Oncol. 2021;23:1231–51. 10.1093/neuonc/noab106. 34185076 10.1093/neuonc/noab106 PMC8328013

[2] Weller M, Van Den Bent M, Tonn JC, Stupp R, Preusser M, Cohen-Jonathan-Moyal E, et al. European Association for Neuro-Oncology (EANO) guideline on the diagnosis and treatment of adult astrocytic and oligodendroglial gliomas. Lancet Oncol. 2017;18:e315–29. 10.1016/S1470-2045(17)30194-8. 28483413 10.1016/S1470-2045(17)30194-8

[3] Van Den Bent MJ, Geurts M, French PJ, Smits M, Capper D, Bromberg JEC, et al. Primary brain tumours in adults. Lancet. 2023;402:1564–79. 10.1016/S0140-6736(23)01054-1. 37738997 10.1016/S0140-6736(23)01054-1

[4] Van Den Bent MJ, French PJ, Brat D, Tonn JC, Touat M, Ellingson BM, et al. The biological significance of tumor grade, age, enhancement, and extent of resection in IDH-mutant gliomas: How should they inform treatment decisions in the era of IDH inhibitors? Neurooncology. 2024;26:1805–22. 10.1093/neuonc/noae107. 10.1093/neuonc/noae107 PMC11449017 38912846

[5] Friedrich M, Bunse L, Wick W, Platten M. Perspectives of immunotherapy in isocitrate dehydrogenase-mutant gliomas. Curr Opin Oncol. 2018;30:368–74. 10.1097/CCO.0000000000000478. 30102604 10.1097/CCO.0000000000000478

[6] Lin MD, Tsai AC-Y, Abdullah KG, McBrayer SK, Shi DD. Treatment of IDH-mutant glioma in the INDIGO era. npj Precis Onc. 2024;8:149. 10.1038/s41698-024-00646-2. 10.1038/s41698-024-00646-2 PMC11258219 39025958

[7] Albert NL, Galldiks N, Ellingson BM, Van Den Bent MJ, Chang SM, Cicone F, et al. PET-based response assessment criteria for diffuse gliomas (PET RANO 1.0): a report of the RANO group. Lancet Oncol. 2024;25:e29–41. 10.1016/S1470-2045(23)00525-9. 38181810 10.1016/S1470-2045(23)00525-9 PMC11787868

[8] Galldiks N, Lohmann P, Albert NL, Tonn JC, Langen K-J. Current status of PET imaging in neuro-oncology. Neurooncol Adv. 2019;1:vdz010. 10.1093/noajnl/vdz010. 32642650 10.1093/noajnl/vdz010 PMC7324052

[9] Müller KJ, Forbrig R, Reis J, Wiegand L, Barci E, Kunte SC, et al. Measurable disease as baseline criterion for response assessment in glioblastoma: A comparison of PET -based (PET RANO 1.0) and MRI-based (RANO) assessments. Neuro-Oncology. Oxford University Press (OUP). 2025; 27:77–88. 10.1093/neuonc/noae208. 10.1093/neuonc/noae208 PMC11726251 39561103

[10] Klemm F, Maas RR, Bowman RL, Kornete M, Soukup K, Nassiri S, et al. Interrogation of the Microenvironmental Landscape in Brain Tumors Reveals Disease-Specific Alterations of Immune Cells. Cell. 2020;181:1643–e166017. 10.1016/j.cell.2020.05.007. 32470396 10.1016/j.cell.2020.05.007 PMC8558904

[11] Zinnhardt B, Pigeon H, Thézé B, Viel T, Wachsmuth L, Fricke IB, et al. Combined PET Imaging of the Inflammatory Tumor Microenvironment Identifies Margins of Unique Radiotracer Uptake. Cancer Res. 2017;77:1831–41. 10.1158/0008-5472.CAN-16-2628. 28137769 10.1158/0008-5472.CAN-16-2628

[12] Unterrainer M, Mahler C, Vomacka L, Lindner S, Havla J, Brendel M, et al. TSPO PET with [18F]GE-180 sensitively detects focal neuroinflammation in patients with relapsing–remitting multiple sclerosis. Eur J Nucl Med Mol Imaging. 2018;45:1423–31. 10.1007/s00259-018-3974-7. 29523925 10.1007/s00259-018-3974-7

[13] Guilarte TR, Rodichkin AN, McGlothan JL, De La Rocha A, Azzam AM. Imaging neuroinflammation with TSPO: A new perspective on the cellular sources and subcellular localization. Pharmacol Ther Elsevier BV. 2022;234:108048. 10.1016/j.pharmthera.2021.108048. 10.1016/j.pharmthera.2021.108048 PMC9107500 34848203

[14] Sridharan S, Raffel J, Nandoskar A, Record C, Brooks DJ, Owen D, et al. Confirmation of Specific Binding of the 18-kDa Translocator Protein (TSPO) Radioligand [18F]GE-180: a Blocking Study Using XBD173 in Multiple Sclerosis Normal Appearing White and Grey Matter. Mol Imaging Biol. 2019;21:935–44. 10.1007/s11307-019-01323-8. 30796709 10.1007/s11307-019-01323-8

[15] Albert NL, Nelwan DV, Fleischmann DF, Quach S, von Rohr K, Kaiser L, et al. Prognostic Value of TSPO PET Before Radiotherapy in Newly Diagnosed IDH-Wild-Type Glioblastoma. J Nucl Med. 2023;64:1519–25. 10.2967/jnumed.122.265247. 37536737 10.2967/jnumed.122.265247 PMC10586482

[16] Weidner L, Lorenz J, Quach S, Braun FK, Rothhammer-Hampl T, Ammer L-M, et al. Translocator protein (18kDA) (TSPO) marks mesenchymal glioblastoma cell populations characterized by elevated numbers of tumor-associated macrophages. acta neuropathol commun. 2023;11:147. 10.1186/s40478-023-01651-5. 37697350 10.1186/s40478-023-01651-5 PMC10496331

[17] Quach S, Holzgreve A, Kaiser L, Unterrainer M, Dekorsy FJ, Nelwan DV, et al. TSPO PET signal using [18F]GE180 is associated with survival in recurrent gliomas. Eur J Nucl Med Mol Imaging. 2023;50:859–69. 10.1007/s00259-022-06006-1. 36329288 10.1007/s00259-022-06006-1 PMC9852133

[18] Ammer L-M, Vollmann-Zwerenz A, Ruf V, Wetzel CH, Riemenschneider MJ, Albert NL, et al. The Role of Translocator Protein TSPO in Hallmarks of Glioblastoma. Cancers MDPI AG. 2020;12:2973. 10.3390/cancers12102973. 10.3390/cancers12102973 PMC7602186 33066460

[19] Menevse AN, Ammer L-M, Vollmann-Zwerenz A, Kupczyk M, Lorenz J, Weidner L, Media LLC et al. 2023 [cited 2025 July 20];11. 10.1186/s40478-023-01550-9.

[20] Wen PY, Van Den Bent M, Youssef G, Cloughesy TF, Ellingson BM, Weller M, et al. RANO 2.0: Update to the Response Assessment in Neuro-Oncology Criteria for High- and Low-Grade Gliomas in Adults. JCO. 2023;41:5187–99. 10.1200/JCO.23.01059. 10.1200/JCO.23.01059 PMC10860967 37774317

[21] Vettermann FJ, Diekmann C, Weidner L, Unterrainer M, Suchorska B, Ruf V, et al. L-type amino acid transporter (LAT) 1 expression in 18F-FET-negative gliomas. EJNMMI Res. 2021;11:124. 10.1186/s13550-021-00865-9. 34905134 10.1186/s13550-021-00865-9 PMC8671595

[22] Albert NL, Unterrainer M, Fleischmann DF, Lindner S, Vettermann F, Brunegraf A, et al. TSPO PET for glioma imaging using the novel ligand 18F-GE-180: first results in patients with glioblastoma. Eur J Nucl Med Mol Imaging. 2017;44:2230–8. 10.1007/s00259-017-3799-9. 28821920 10.1007/s00259-017-3799-9

[23] Law I, Albert NL, Arbizu J, Boellaard R, Drzezga A, Galldiks N, et al. Joint EANM/EANO/RANO practice guidelines/SNMMI procedure standards for imaging of gliomas using PET with radiolabelled amino acids and [18F]FDG: version 1.0. Eur J Nucl Med Mol Imaging. Springer Sci Bus Media LLC. 2019;46:540–57. 10.1007/s00259-018-4207-9. 10.1007/s00259-018-4207-9 PMC6351513 30519867

[24] Quach S, Holzgreve A, Kaiser L, Unterrainer M, Dekorsy FJ, Nelwan DV, et al. TSPO PET signal using [18F]GE180 is associated with survival in recurrent gliomas. Eur J Nucl Med Mol Imaging. 2023;50:859–69. 10.1007/s00259-022-06006-1. 36329288 10.1007/s00259-022-06006-1 PMC9852133

[25] Unterrainer M, Fleischmann DF, Vettermann F, Ruf V, Kaiser L, Nelwan D, et al. TSPO PET, tumour grading and molecular genetics in histologically verified glioma: a correlative 18F-GE-180 PET study. Eur J Nucl Med Mol Imaging. 2020;47:1368–80. 10.1007/s00259-019-04491-5. 31486876 10.1007/s00259-019-04491-5

[26] Bartos LM, Kirchleitner SV, Kolabas ZI, Quach S, Beck A, Lorenz J, et al. Deciphering sources of PET signals in the tumor microenvironment of glioblastoma at cellular resolution. Sci Adv. 2023;9:eadi8986. 10.1126/sciadv.adi8986. 37889970 10.1126/sciadv.adi8986 PMC10610915

[27] Kaiser L, Quach S, Zounek AJ, Wiestler B, Zatcepin A, Holzgreve A, et al. Enhancing predictability of IDH mutation status in glioma patients at initial diagnosis: a comparative analysis of radiomics from MRI, [18F]FET PET, and TSPO PET. Eur J Nucl Med Mol Imaging. 2024;51:2371–81. 10.1007/s00259-024-06654-5. 38396261 10.1007/s00259-024-06654-5 PMC11178656

[28] Quach S, Holzgreve A, Von Baumgarten L, Niyazi M, Unterrainer M, Thon N, et al. Increased TSPO PET signal after radiochemotherapy in IDH-wildtype glioma—indicator for treatment-induced immune activation? Eur J Nucl Med Mol Imaging. 2022;49:4282–3. 10.1007/s00259-022-05844-3. 35610517 10.1007/s00259-022-05844-3 PMC9525328

[29] Kaiser L, Holzgreve A, Quach S, Ingrisch M, Unterrainer M, Dekorsy FJ, et al. Differential Spatial Distribution of TSPO or Amino Acid PET Signal and MRI Contrast Enhancement in Gliomas. Cancers. 2021;14:53. 10.3390/cancers14010053. 35008218 10.3390/cancers14010053 PMC8750092

[30] Raman F, Mullen A, Byrd M, Bae S, Kim J, Sotoudeh H, et al. Evaluation of RANO Criteria for the Assessment of Tumor Progression for Lower-Grade Gliomas. Cancers. 2023;15:3274. 10.3390/cancers15133274. 37444384 10.3390/cancers15133274 PMC10340202

[31] Jansen NL, Schwartz C, Graute V, Eigenbrod S, Lutz J, Egensperger R, et al. Prediction of oligodendroglial histology and LOH 1p/19q using dynamic [18F]FET-PET imaging in intracranial WHO grade II and III gliomas. Neurooncology. 2012;14:1473–80. 10.1093/neuonc/nos259. 10.1093/neuonc/nos259 PMC3499015 23090986

[32] Galldiks N, Werner J-M, Stetter I, Puhr HC, Nakuz TS, Stoffels G, et al. Evaluation of early metabolic changes following vorasidenib using FET PET in patients with *IDH* -mutant gliomas. Neuro-Oncology Adv. 2024;6:vdae210. 10.1093/noajnl/vdae210. 10.1093/noajnl/vdae210 PMC11683584 39737092

[33] Albert NL, Furtner J, van den Bent MJ, Preusser M. The potential of amino acid PET imaging for prediction and monitoring of vorasidenib response in IDH-mutant gliomas. Neuro Oncol. 2024;26:403–6. 10.1093/neuonc/noad240. 38070497 10.1093/neuonc/noad240 PMC10911996

[34] Mair MJ, Werner J-M, Weller J, Barci E, Katzendobler S, Isakaj J, et al. Prognostic stratification of newly diagnosed *IDH-* mutant gliomas by [18F]fluoroethyltyrosine and [11 C]methionine PET – a retrospective, bicentric cohort study. Neurooncology. 2025;noaf196. 10.1093/neuonc/noaf196. 10.1093/neuonc/noaf196 PMC12916739 40856188

[35] Wollring MM, Werner J-M, Ceccon G, Lohmann P, Filss CP, Fink GR, et al. Clinical applications and prospects of PET imaging in patients with IDH-mutant gliomas. J Neurooncol Springer Sci Bus Media LLC. 2023;162:481–8. 10.1007/s11060-022-04218-x. 10.1007/s11060-022-04218-x PMC10227162 36577872

[36] Unterrainer M, Fleischmann DF, Vettermann F, Ruf V, Kaiser L, Nelwan D, et al. TSPO PET, tumour grading and molecular genetics in histologically verified glioma: a correlative 18F-GE-180 PET study. Eur J Nucl Med Mol Imaging Springer Sci Bus Media LLC. 2020;47:1368–80. 10.1007/s00259-019-04491-5. 10.1007/s00259-019-04491-5 31486876

[37] Vettermann FJ, Harris S, Schmitt J, Unterrainer M, Lindner S, Rauchmann B-S, et al. Impact of TSPO Receptor Polymorphism on [18F]GE-180 Binding in Healthy Brain and Pseudo-Reference Regions of Neurooncological and Neurodegenerative Disorders. Life. 2021;11:484. 10.3390/life11060484. 34073557 10.3390/life11060484 PMC8229996

[38] Feeney C, Scott G, Raffel J, Roberts S, Coello C, Jolly A, et al. Kinetic analysis of the translocator protein positron emission tomography ligand [18F]GE-180 in the human brain. Eur J Nucl Med Mol Imaging. 2016;43:2201–10. 10.1007/s00259-016-3444-z. 27349244 10.1007/s00259-016-3444-z PMC5047949

[39] Zanotti-Fregonara P, Pascual B, Rizzo G, Yu M, Pal N, Beers D, et al. Head-to-Head Comparison of^11^ C-PBR28 and^18^ F-GE180 for Quantification of the Translocator Protein in the Human Brain. J Nucl Med. 2018;59:1260–6. 10.2967/jnumed.117.203109. 29348317 10.2967/jnumed.117.203109

