# Supplementary material for: The prognostic significance of TSPO-PET imaging in IDH-mutant glioma: a single-center, retrospective study
Source: Eur J Nucl Med Mol Imaging. 2026 May 30;53(10):5733–44. doi: 10.1007/s00259-026-07926-y (PMC13421190; doi:10.1007/s00259-026-07926-y)
Supplement: Supplementary file 1 — Supplementary Material 1 [file 259_2026_7926_MOESM1_ESM.docx]

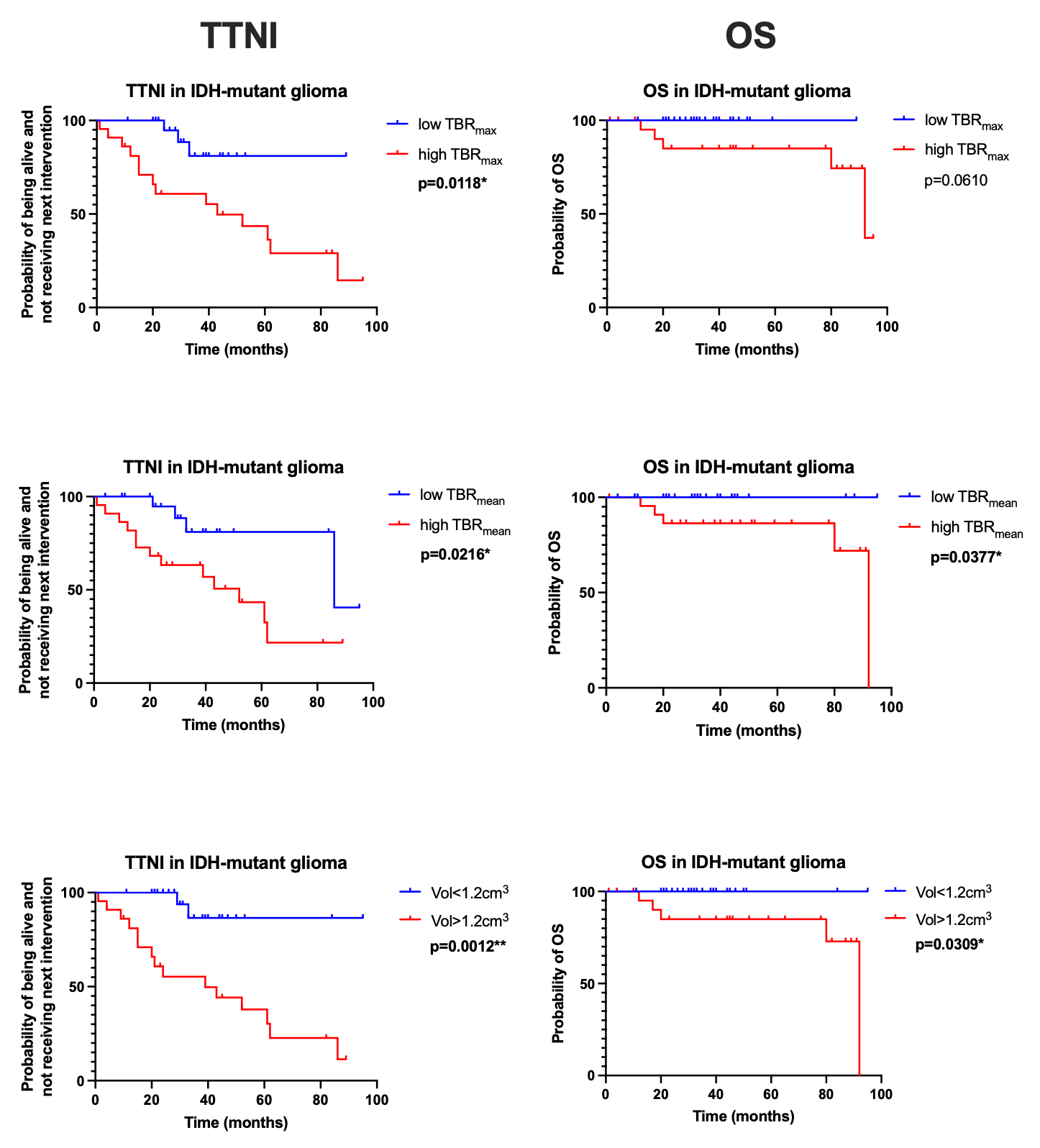


**Supplemental Figure 1** Kaplan-Meier estimation of TTNI and OS based on additional TSPO-PET parameters (n=46): Stratification for TBR_max_, TBR_mean_, and PET-positive volume are illustrated. We identified significant associations between low vs. high TBR_mean_ with TTNI (p=0.0216), and OS (p=0.0377), as well as for TBR_max_ and TTNI (p=0.0118). Based on the TSPO-PET-positive volume, TTNI and OS were significantly different (p=0.0012, p=0.0309, Fig. 4).
