# Supplementary material for: The prognostic significance of TSPO-PET imaging in IDH-mutant glioma: a single-center, retrospective study
Source: Eur J Nucl Med Mol Imaging. 2026 May 30;53(10):5733–44. doi: 10.1007/s00259-026-07926-y (PMC13421190; doi:10.1007/s00259-026-07926-y)
Supplement: Supplementary file 2 — Supplementary Material 2 [file 259_2026_7926_MOESM2_ESM.docx]

**
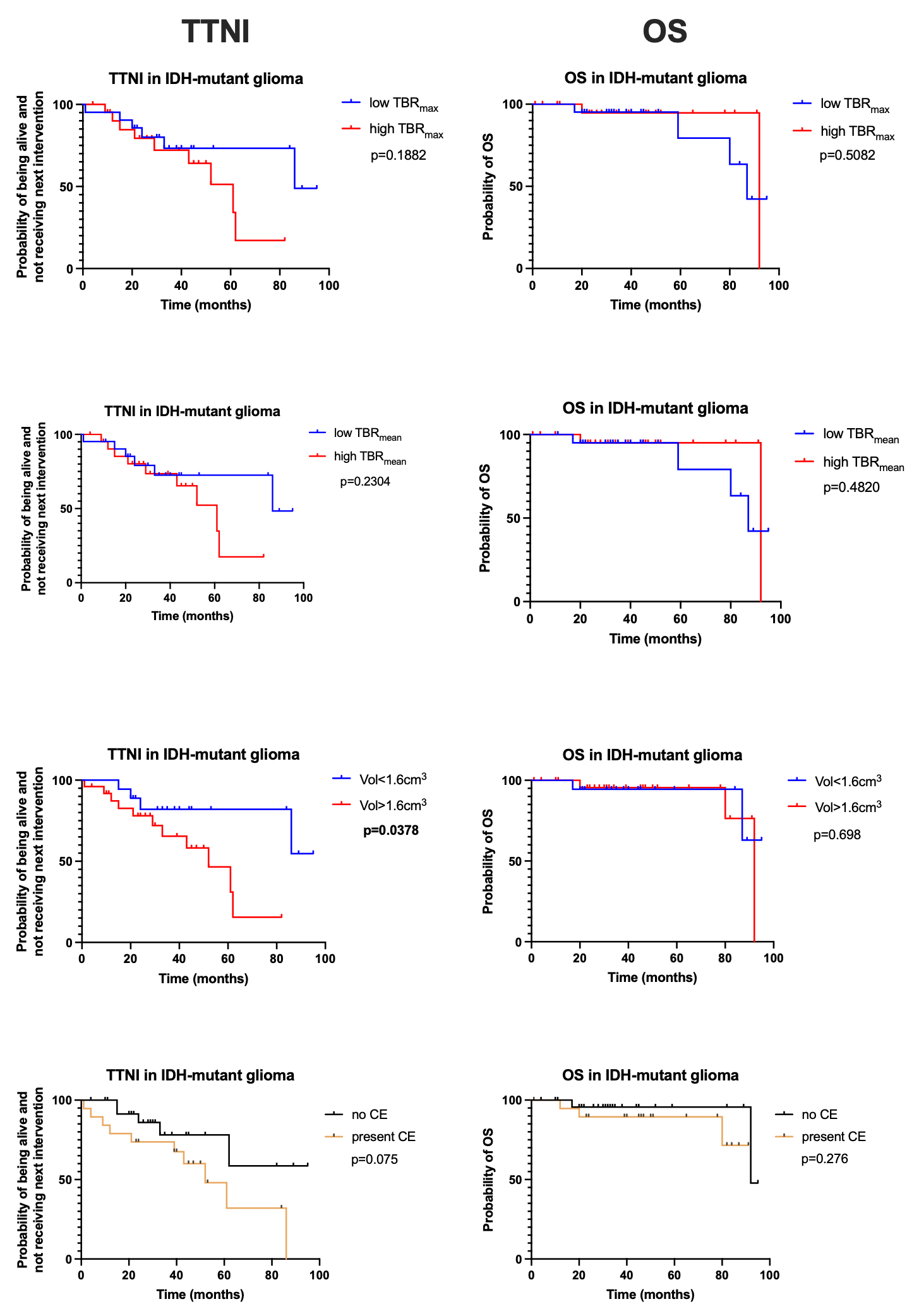
**

**Supplemental Figure 2** Kaplan-Meier estimation of PFS, TTNI and OS in 44 patients, stratified for SUV_max_, TBR_max_, TBR_mean_, and FET-PET-positive volume. Presence of CE on MRI is also illustrated. For FET-PET, only PET-positive volume correlated with TTNI (p=0.0378), whereas other parameters (TBR_mean_, TBR_max_) and contrast enhancement on MRI showed no significant associations.
