# Supplementary material for: The prognostic significance of TSPO-PET imaging in IDH-mutant glioma: a single-center, retrospective study
Source: Eur J Nucl Med Mol Imaging. 2026 May 30;53(10):5733–44. doi: 10.1007/s00259-026-07926-y (PMC13421190; doi:10.1007/s00259-026-07926-y)
Supplement: Supplementary file 3 — Supplementary Material 3 [file 259_2026_7926_MOESM3_ESM.docx]

**
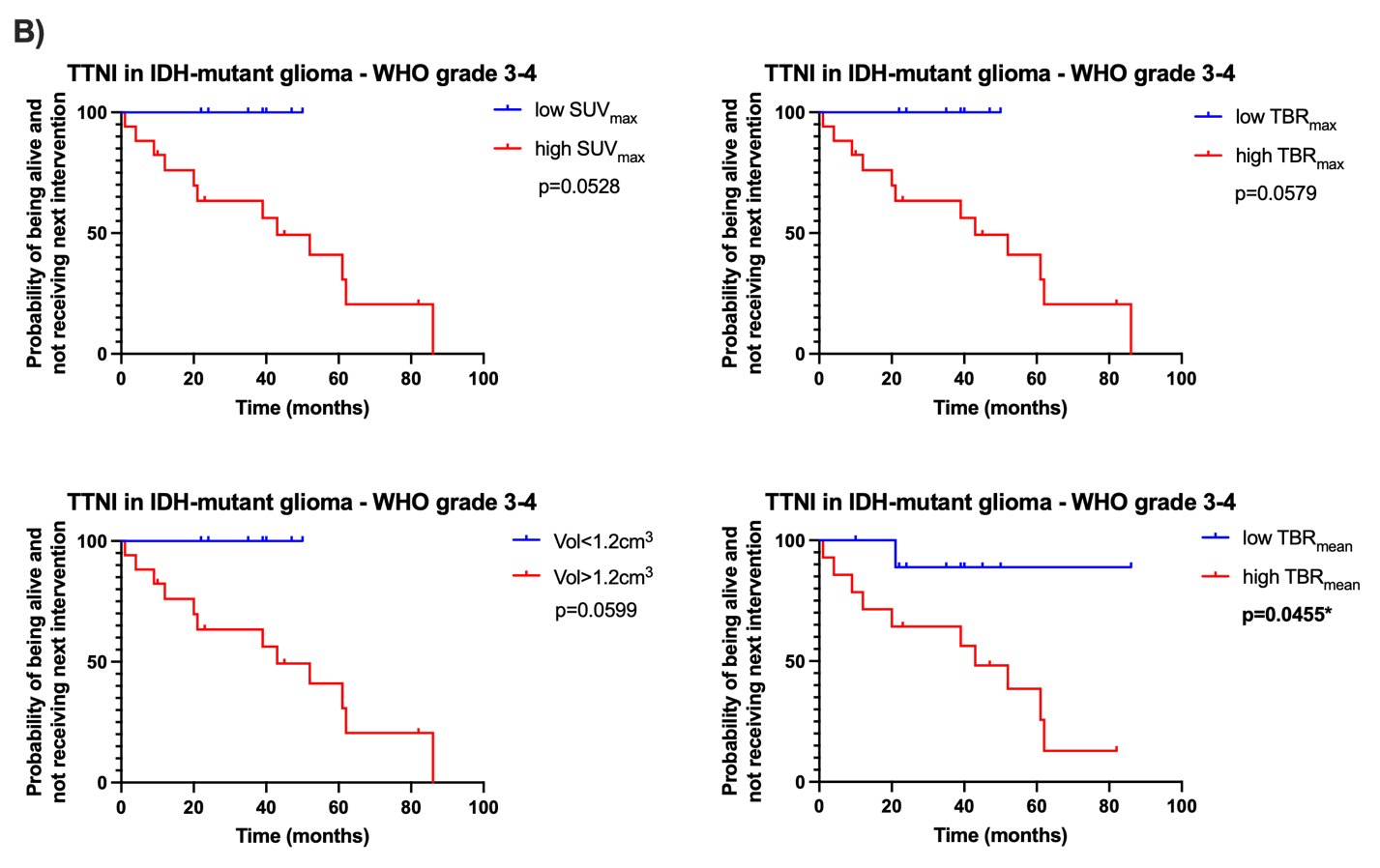

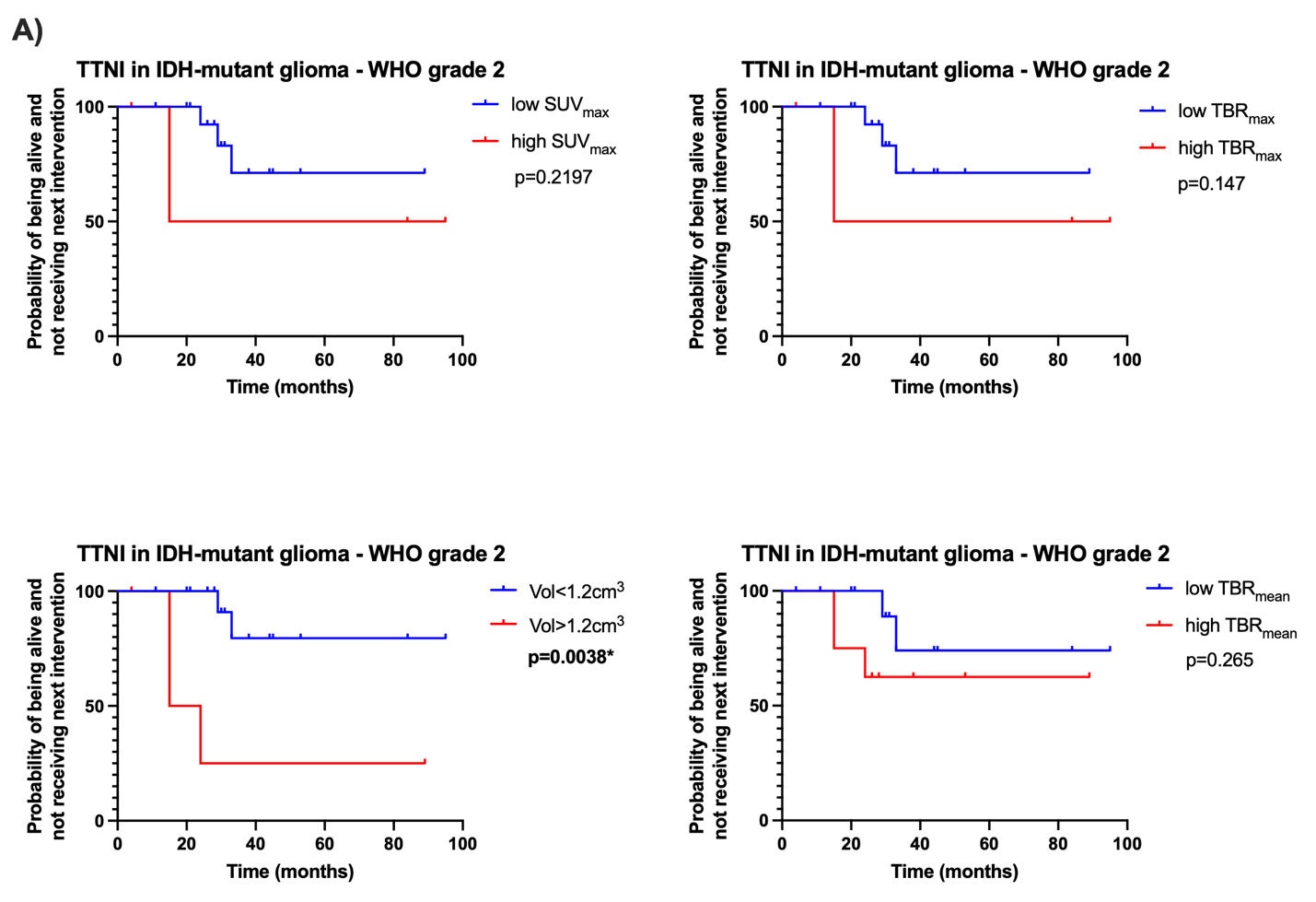
Supplemental figure 3** Kaplan-Meier analysis of TTNI according to [^18^F]GE180-PET parameters in WHO grade 2 (n=22) and WHO grade 3-4 IDH-mutant glioma (n=24)

A) In WHO grade 2, higher [^18^F]GE180-PET-positive volume was significantly associated with shorter TTNI (p=0.0038), whereas tracer uptake intensity was not significant. B) In WHO grade 3-4, higher TBR_mean_ on [^18^F]GE180-PET was significantly associated with shorter TTNI (p=0.0455).
