# Supplementary material for: The prognostic significance of TSPO-PET imaging in IDH-mutant glioma: a single-center, retrospective study
Source: Eur J Nucl Med Mol Imaging. 2026 May 30;53(10):5733–44. doi: 10.1007/s00259-026-07926-y (PMC13421190; doi:10.1007/s00259-026-07926-y)
Supplement: Supplementary file 4 — Supplementary Material 4 [file 259_2026_7926_MOESM4_ESM.docx]

| Imaging characteristics | WHO grade 2 | WHO grade 3-4 | *p-*value |
| --- | --- | --- | --- |
| T2 volume [cm^3^] | 15 (9-32) | 22 (15-36) 0.938 | |
| CE-T1 volume [cm^3^] | 0 (0-0.2) | 0.65 (0.7-2.2) ***0.001** | |
| [^18^F]FET TBR_max_ | 1.8 (1.5-2.4) | 2.8 (2.0-3.8) ***0.006** | |
| [^18^F]FET volume [cm^3^] | 0.2 (0-4.5) | 1.5 (12-22) ***0.004** | |
| [^18^F]GE-180 TBR_max_ | 1.8 (1.6-2.3) | 2.4 (1.9-3.0) ***0.008** | |
| [^18^F]GE-180 volume [cm^3^] | 0.25 (0.1-2.0) | 5.9 (0.44-13) ***0.003** | |

**Supplemental Table 1** Imaging characteristics in WHO grade 2 vs. 3-4 cases.

| [^18^F]GE-180 PET metrics | MAB/LAB  n=18 | HAB  n=20 | *p-*value |
| --- | --- | --- | --- |
| Background activity | 0.38 (0.37-0.41) | 0.38 (0.34-0.42) 0.567 | |
| SUV_mean_ | 0.64 (0.54-0.95) | 0.66 (0.55-0.78) 0.745 | |
| SUV_max_ | 0.98 (0.57-1.6) | 0.67 (0.56-1.2) 0.429 | |
| TBR_mean_ | 1.7 (1.2-2.3) | 1.7 (1.5-2.0) 0.857 | |
| TBR_max_ | 2.4 (1.5-3.1) | 2.0 (1.6-2.9) 0.587 | |
| PET-positive volume [cm^3^] | 1.1 (0.2-7.3) | 1.4 (0.11-7.9) 0.857 | |

**Supplemental Table 2** TSPO-PET metrics compared to TSPO binding status, missing status in n=8 patients.
